# A human Phase I/IIa malaria challenge trial of a polyprotein malaria vaccine

**DOI:** 10.1016/j.vaccine.2011.03.083

**Published:** 2011-10-06

**Authors:** David W. Porter, Fiona M. Thompson, Tamara K. Berthoud, Claire L. Hutchings, Laura Andrews, Sumi Biswas, Ian Poulton, Eric Prieur, Simon Correa, Rosalind Rowland, Trudie Lang, Jackie Williams, Sarah C. Gilbert, Robert E. Sinden, Stephen Todryk, Adrian V.S. Hill

**Affiliations:** a Centre for Clinical Vaccinology and Tropical Medicine, Churchill Hospital, Old Road, Oxford, OX3 7LJ, UK; b Jenner Institute, University of Oxford, Old Road Campus Research Building, Oxford, OX3 7DQ, UK; c Medical Research Council (MRC) Laboratories, Atlantic Boulevard, Fajara, PO Box 273 Banjul, Gambia; d Division of Entomology, Walter Reed Army Institute of Research, Silver Spring, Maryland, MD 20910, USA; e Infection and Immunity Section, Biology Department, Sir Alexander Fleming Building, Imperial College, London, SW7 2AZ, UK

**Keywords:** Malaria, Vaccine, Heterologous prime-boost

## Abstract

We examined the safety, immunogenicity and efficacy of a prime-boost vaccination regime involving two poxvirus malaria subunit vaccines, FP9-PP and MVA-PP, expressing the same polyprotein consisting of six pre-erythrocytic antigens from *Plasmodium falciparum*.

Following safety assessment of single doses, 15 volunteers received a heterologous prime-boost vaccination regime and underwent malaria sporozoite challenge. The vaccines were safe but interferon-γ ELISPOT responses were low compared to other poxvirus vectors, despite targeting multiple antigens. There was no vaccine efficacy as measured by delay in time to parasitaemia. A number of possible explanations are discussed, including the very large insert size of the polyprotein transgene.

## 1 Introduction

*Plasmodium falciparum* is responsible for an enormous worldwide burden of human disease, causing an estimated 200–500 million cases of clinical disease and 1 million deaths each year [1,2], most of this occurring in sub-Saharan Africa. Two billion people are thought to live in areas at significant risk of malaria [1]. However, it is clear from irradiated sporozoite studies in humans that it is possible to induce effective and relatively durable immunity against *P. falciparum* and that this can be strain-transcending [3]. Despite this proof of principle, there remains no currently available malaria vaccine.

A number of vaccine strategies are being explored at present, most of which focus on one or very few parasite antigens. In contrast, the poxvirus-vectored vaccines used in this study were constructed to encode the entire sequence of six separate *P. falciparum* proteins expressed at the pre-erythrocytic stage yielding a 3240 amino-acid long ‘polyprotein’ [4]. This strategy aimed to generate a broad cellular immune response directed against a variety of pre-erythrocytic parasite antigens, rather than a strong but narrow response. The proteins were selected using immunogenicity data from humans living in malaria endemic areas and from responses against irradiated sporozoites. This approach is supported by the fact that although the immunodominant circumsporozoite (CS) protein response plays an important role in the protective effect of irradiated sporozoite vaccination in mice, protection can still be induced when CS is removed as an immune target [5]. Protection may then be achieved with the combination of modest responses against a number of parasite proteins. A broader response could also reduce the risk of parasite immune escape and be effective against a variety of parasite strains and across varying Human Leukocyte Antigen (HLA) types. Significant humoral responses were not expected or examined for in this study.

The viral vectors fowlpox strain FP9 and modified vaccinia virus Ankara (MVA) have an excellent safety record in humans [6–8], are capable of inducing powerful T-cell responses [9,10] and have been shown to induce protection against malaria in mice [10] and in humans [7]. Both have been engineered to express the polyprotein construct (FP9-PP and MVA-PP). When evaluated in mice, FP9-PP was specifically shown to induce IFNγ-secreting T cells by ELISPOT against each of the six vaccine antigens and heterologous prime-boost vaccination induced liver-stage antigen 1 (LSA-1) tetramer positive CD8 T-cells that demonstrated cytotoxic activity [4]. The importance of IFNγ has been shown by its ability to inhibit development of exoerythrocytic parasite forms within hepatocytes [11].

This study examines the safety, immunogenicity and challenge efficacy of these vaccines when administered to healthy human volunteers intradermally, four weeks apart in two different prime-boost regimes.

## 2 Materials and methods

### 2.1 Volunteers and recruitment

Healthy malaria naïve adults aged 18–50 years old were recruited from April 2006 to November 2006 from the Oxford area in the UK. Screening, vaccination and all study visits except for the sporozoite challenge day itself were carried out at the Centre for Clinical Vaccinology and Tropical Medicine, University of Oxford, Churchill Hospital, Oxford, UK. The malaria challenge took place at the insectary of the Alexander Fleming Building, Imperial College, London, UK.

Key study exclusion criteria included: abnormal baseline haematology or biochemistry; evidence of hepatitis B, C or HIV infection; history of immunosuppressive medication or immunodeficiency; previous history of malaria; malaria chemoprophylaxis within five months (for challenge volunteers); travel to a malaria endemic region within six months; or history or evidence of a significant physical or psychiatric disorder.

### 2.2 Funding, ethical and regulatory approval

This study was principally funded by the European Malaria Vaccine Initiative (EMVI) now European Vaccine Initiative (EVI) and sponsorship responsibilities were shared through delegation between EMVI and the University of Oxford. The trial protocol and associated documents were reviewed and approved as two studies by the Oxfordshire National Health Service Research Ethics Committee A (OxREC A, reference numbers 04/Q1604/93 and 06/Q1604/55) and by the Medicines and Healthcare products Regulatory Agency (MHRA, EudraCT numbers 2004-002424-17 and 2006-000629-67). Recombinant vaccine use was authorised by the Genetic Modification Safety Committee (GMSC) of the Oxford Radcliffe Hospitals NHS Trust (reference number GM462.04.21).

All volunteers gave written informed consent before enrolment and the study was conducted according to the principles of the Declaration of Helsinki and in accordance with Good Clinical Practice (GCP). External study monitoring was provided by Appledown Clinical Research.

### 2.3 Study design

Study groups 1–5 (*n* = 3 each) were single dose-escalation groups with the following doses: FP9-PP at 1 × 10^8^ plaque-forming units (pfu), MVA-PP at 1 × 10^8^ pfu, FP9-PP at 2 × 10^8^ pfu, MVA-PP at 2 × 10^8^ pfu and MVA-PP at 5 × 10^8^ pfu respectively. Volunteers in groups 6 and 7 (planned *n* = 10 each) received the heterologous prime-boost vaccine regimes ‘FFM’ or ‘MMF’ respectively. ‘FFM’ refers to the sequence of FP9-PP/FP9-PP/MVA-PP with each vaccination one month apart. ‘MMF’ refers to the equivalent sequence of MVA-PP/MVA-PP/FP9-PP. Doses were 1, 1 and 2 × 10^8^ pfu for first, second and third vaccinations for both groups 6 and 7. Control volunteers (*n* = 6) were recruited to undergo malaria challenge without vaccination to confirm the infective efficacy of the sporozoite challenge. Vaccine follow-up visits for groups 1–7 were on days 2, 7 and 28 following each vaccination with additional visits on day 90 (groups 1–5) and day 150 after first vaccination (groups 6 and 7). In addition, all challengees were seen regularly during the three weeks following challenge (see *sporozoite challenge* below) and then 35 and 150 days following challenge. Blood was collected regularly for safety assessments and immunogenicity.

### 2.4 Vaccines and ‘polyprotein’ insert

FP9-PP and MVA-PP were manufactured according to Good Manufacturing Practice (GMP) regulations by Impfstoffwerk Dessau-Tornau (IDT, Roßlau, Germany). The polyprotein vaccine insert (‘L3SEPTL’) has been fully described before [4]. It contains six pre-erythrocytic malaria antigens linked together in a single protein (from N to C terminus): liver stage antigen 3 (LSA3) [12], sporozoite threonine and asparagine rich protein (STARP) [13], exported protein-1 (Exp1) [14], Pfs16 [15], thrombospondin-related adhesion protein (TRAP) [16] and liver stage antigen-1 (LSA1) [17]. All except possibly Pfs16 are pre-erythrocytic antigens; LSA3, Exp1 and STARP are also expressed by blood-stage parasites and Pfs16 is also a sexual-stage antigen [4].

Vaccines were stored at the trial site at −80 °C and thawed shortly before administration. Each dose was given intradermally into the skin overlying the deltoid muscle of the upper arm. Doses were divided equally between both arms. Vaccine sites were temporarily covered with an absorbent dressing which was removed when the vaccine sites were reassessed approximately 30 min later.

### 2.5 Adverse events

Volunteers were asked to complete study diary cards for the first seven days after vaccination, beginning with the evening of the vaccination day. These recorded local reactions (pain, redness, swelling, itching, warmth and scaling) and systemic symptoms (oral temperature, feverishness, myalgia, arthralgia, nausea or vomiting, lethargy, headache and malaise). Temperature was measured with an oral digital thermometer (Servoprax GmbH) supplied by the investigators and redness and swelling were recorded as maximal diameters (ensuring the measurement passed through the puncture site). On each clinic attendance the investigators independently collected the same measurements. Adverse events (AEs) were recorded at each clinic visit in response to direct questioning, self-reporting on volunteer diary cards and examination of the vaccine site at each attendance by the investigators.

Severity scales used for grading are shown in Online Table A. AEs were judged as either unrelated or possibly, probably or definitely related to vaccination by the investigator, taking into account the symptoms and time since vaccination. All AEs were followed until resolution where possible. If the study ended before resolution, attempts were made to determine outcome by contacting the volunteer and/or general practitioner. The data presented here includes all AEs, even if a volunteer subsequently dropped out of the study. Where an AE stopped and restarted within 30 days of vaccination it has only been reported once in these results, but durations have been summed. AE durations have been rounded up to the nearest day.

### 2.6 Sporozoite challenge

Volunteers underwent *P. falciparum* sporozoite challenge at Imperial College, London two weeks after the final vaccination. They each received bites from five mosquitoes subsequently confirmed to have more than 100 sporozoites per paired salivary gland. *Anopheles stephensi* mosquitoes were infected with the chloroquine-sensitive 3D7 strain of the parasite at the Walter Reed Army Institute of Research (WRAIR), Maryland, US and reared in the laboratory as previously described [18]. Volunteers began attending clinic for malaria screening from the evening of day 6 after infection. At each visit they were questioned about possible symptoms, had their temperature, pulse and blood pressure measured and gave blood for both thick film microscopy and PCR for malaria parasites. This process was repeated twice daily from day 7 to day 14 and then once daily from days 15 to 21, or until diagnosis. Two experienced microscopists examined a minimum of 200 high power fields (100× objective) for parasite ring forms on each sample. A diagnosis of malaria was made as soon as one or more viable parasites were seen on a volunteer's slide. Oral anti-malarial treatment was commenced on diagnosis as an outpatient with oral Riamet^®^ (Novartis, 20 mg artemether with 120 mg lumefantrine) given at diagnosis and then approximately 8, 24, 36 and 48 h later. Artemether combination therapy was chosen in line with World Health Organisation recommendations on the treatment of uncomplicated malaria. Volunteers returned for repeat blood film examinations daily after treatment commenced until two consecutive negative films had been seen.

### 2.7 PCR and parasite growth rate analysis

Quantitative real-time PCR was performed at challenge baseline and at all post-challenge visits until treatment commenced using a method described previously [19]. Clinicians, volunteers and staff performing other assays were blinded to the PCR results during the study. Data was adjusted using a standard curve derived from counted cultured parasites in whole blood to give the number of parasites per mL of blood.

The PCR data was also used to estimate overall growth rates of blood stage parasites during the challenge for each volunteer and to back-calculate a starting number of merozoites emerging into the blood (around day 6–7) and hence an estimate of the number of infected hepatocytes responsible for initial seeding of blood-stage parasite forms. The methods employed are based on an iterative adjustment model to derive a best fit curve to the measured data, as described [20].

### 2.8 ELISPOT assay

*Ex vivo* IFNγ-ELISPOTs were carried out broadly as described [21]. Briefly, fresh heparinised blood was separated using Lymphoprep (Nycomed Pharma), washed and resuspended in RPMI-1640 (Sigma–Aldrich) containing 10% heat-inactivated fetal bovine serum (BioSera), 100 U/mL penicillin, 100 μg/mL streptomycin and 2 mM l-glutamine (both Invitrogen) and PBMC were counted using an automated CasyCounter TT (Innovatis AG). PBMC were plated in duplicate wells at 0.4 million per well on MultiScreen 96-well HPVDF filtration plates (MAIPS4510, Millipore) after coating overnight at 4 °C with 10 μg/mL of anti-IFNγ (1-D1K, Mabtech) and blocking with the supplemented medium described above. Cells were incubated (37 °C, 5% CO_2_) for 18–20 h with positive (phytohaemagglutinin 10 μg/mL, Sigma) or negative (supplemented medium) controls or peptide pools consisting of up to 32 peptides (each 20mers overlapping by 10, final concentration 10 μg/mL/peptide). Plates were developed using biotin–streptavidin–ALP (Mabtech) with the addition of a chromogenic substrate (BioRad). Spots were counted using an ELISPOT reader and associated software (both Autoimmun Diagnostika). Final counts were expressed as sfu/million PBMC after averaging duplicate well counts and subtracting background. For larger proteins, responses from multiple peptide pools were summed to give the response against the whole protein.

### 2.9 Data analysis

Data analysis was carried out using Microsoft Excel^®^, GraphPad Prism^®^ and STATACorp STATA^®^ with Kaplan-Meier analysis in SPSS^®^.

## 3 Results

### 3.1 Volunteer recruitment and group allocation

A total of 34 volunteers passed screening and were enrolled into study groups 1–7 between April and November 2006. Volunteer demographics are shown in Table 1. Fifteen volunteers received one vaccination each in the dose-escalation groups 1–5 (*n* = 3 per group). Nineteen volunteers were enrolled into the prime-boost vaccination groups 6 (or ‘FFM’ receiving the vaccine sequence FP9-PP/FP9-PP/MVA-PP, *n* = 9) and 7 (‘MMF’, *n* = 10). Three volunteers subsequently withdrew (one from the FFM group due to a pre-existing condition not revealed at screening and two from the MMF group due to unforeseen changes to work and travel plans). All available data has been included in the analysis for these volunteers. Fifteen of the 16 volunteers completing the prime-boost vaccination study subsequently volunteered to enter the separate but linked challenge study. They were joined by six newly-recruited unvaccinated malaria-naïve challenge control volunteers.

### 3.2 Vaccination with FP9-PP and MVA-PP is safe and well-tolerated

**Fig. 1:**
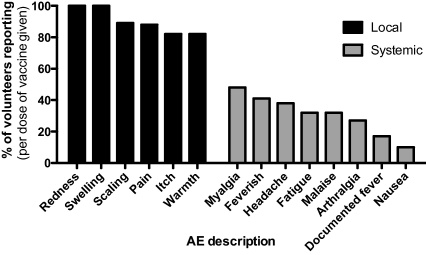
Frequency of vaccine-related solicited AEs in all groups. The proportion of volunteers reporting each solicited AE within one month of vaccination is shown, per vaccine dose (total doses = 68). The data is a combination of AEs from single dose and prime-boost vaccination groups. ‘Local’ AEs occurred at or around the vaccination site.

No serious adverse events (SAEs) occurred during the study. Of 717 adverse events (AEs) recorded during the entire vaccination phase, 577 (81%) were judged probably or definitely related to vaccination (termed ‘vaccine-related’ from here on). Of these, 562 (97%) were AEs anticipated from previous studies of these vaccine vectors about which volunteers were specifically asked at each visit (solicited AEs, Fig. 1). The majority of all AEs reported during the vaccination phase were mild, with only 1 (0.1%) graded severe and 8% moderate in severity. The severe AE was local swelling at the vaccine site. This AE was graded ‘severe’ on the basis of the volunteer diary card readings, which were >50 mm on the day following vaccination only (90 and 80 mm for left and right arms respectively). By day 2 volunteer measurements were 34 and 28 mm and clinic measurements 20 and 12 mm (left and right arms respectively). The volunteer reported that the total duration of swelling was 13 days.

**Fig. 2:**
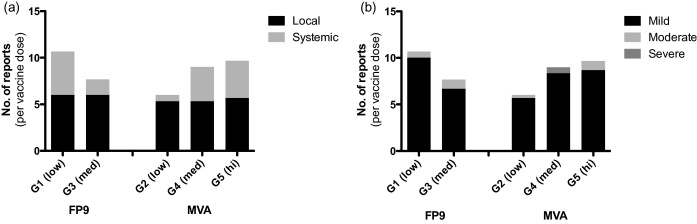
Frequency of vaccine-related AEs in single dose groups. For each dose of vaccine given (*n* = 15), the mean number of AE reports recorded within one month of each vaccine dose is shown by (a) AE site or (b) severity of AE. Multiple reports of the same AE for the same volunteer have only been counted once. Vaccine doses were 1 × 10^8^, 2 × 10^8^ or 5 × 10^8^ pfu for low, med and hi groups respectively.

**Fig. 3:**
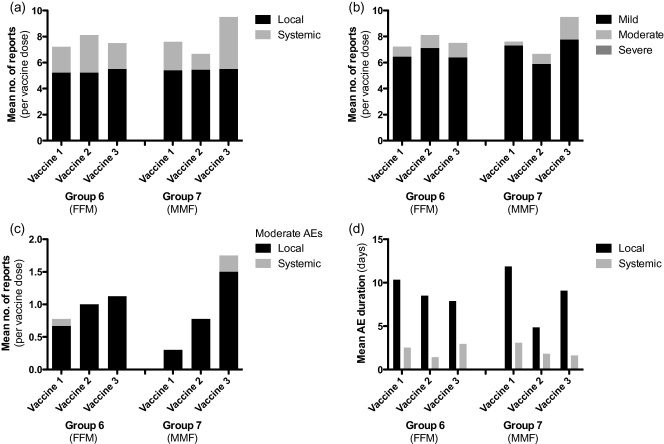
Frequency and duration of vaccine-related AEs in prime-boost groups. For each dose of vaccine given (*n* = 68), the mean number of AE reports recorded within one month of each vaccine dose is shown by (a) AE site or (b) severity of AE. (c) Shows moderate AEs only and (d) the mean duration in days of related AEs beginning within one month of vaccination. Multiple reports of the same AE for the same volunteer and vaccine dose have only been counted once, but durations have been summed. Vaccine doses were 1 × 10^8^ for vaccine 1 (prime) and 2 × 10^8^ for vaccines 2 and 3 (boosts) for both groups.

Of vaccine-related AEs (detailed in Online Table B), 394 (68%) were local to the vaccine site and 183 (32%) were systemic. The median AE duration (and interquartile range, IQR) was 7 (3–12) and 2 (1–2) days for local and systemic vaccine-related AEs respectively. As expected, local vaccine responses (such as pain, redness, swelling and local tenderness) occurred with almost every vaccine dose. The median duration (and IQR) of pain was 2 (1–3.25) days and most (88.2%) were mild. Systemic responses (e.g. headache, myalgia and tiredness) occurred frequently after vaccination (Fig. 1). Myalgia was most common, reported by 48% of volunteers. For the single vaccine dose-escalation groups 1–5, the frequency of local AEs did not alter as dose increased, but more systemic AEs (mostly mild in severity) were seen with increasing dose in MVA vaccinated volunteers (Fig. 2). The frequency of local AEs also varied little with successive vaccinations in the three-dose heterologous prime-boost groups FFM and MMF, but the proportion of AEs graded moderate increased with successive doses in the MMF group (Fig. 3). There was no clear trend in AE duration during vaccination in these groups (Fig. 3d).

Eleven volunteers (32%) had at least one blood result falling outside the study reference ranges during follow up, but none of these were associated with clinical symptoms and only two warranted referral to the general practitioner for repeat testing or investigation (mild hyperbilirubinaemia at 28 μmol/L and a low haemoglobin of 9.8 g/dL which resolved at repeat testing).

### 3.3 Single dose immunogenicity and dose selection for prime-boost groups

Three doses of MVA-PP and two doses of FP9-PP were assessed in single-dose small groups (*n* = 3), primarily for safety, before deciding on doses to be used in the larger prime-boost groups.

**Fig. 4:**
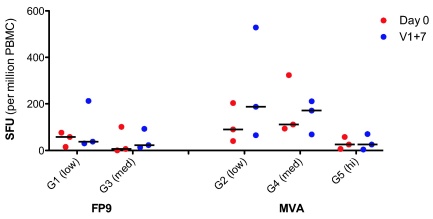
Single-dose group immunogenicity. *Ex vivo* IFNγ-ELISPOT responses to the whole vaccine insert (‘L3SEPTL’) at baseline and 7 days following vaccination (‘D0’ and ‘V1+7’ respectively). Responses are displayed as spot forming units (sfu) per million PBMC. Horizontal lines represent the median.

Immunogenicity for these groups was low, as expected in the absence of a booster dose, but pre-vaccination responses were also relatively high (Fig. 4). For MVA-PP there was a suggestion that immunogenicity was lower at the high dose (5 × 10^8^ pfu).

In deciding the dose to be used in the prime-boost groups, the following factors were considered: firstly, although all doses appeared safe, the frequency of systemic AEs was higher with increasing MVA-PP dose; secondly, there was no clear dose advantage for MVA-PP at high dose; and thirdly, the possibility of encountering anti-vector immunity cross-reactive between the different poxviruses. It was therefore decided that for each of the prime-boost groups, the low vaccine dose (1 × 10^8^ pfu) would be used to prime and the intermediate dose (2 × 10^8^ pfu) to boost.

### 3.4 Modest prime-boost immunogenicity

**Fig. 5:**
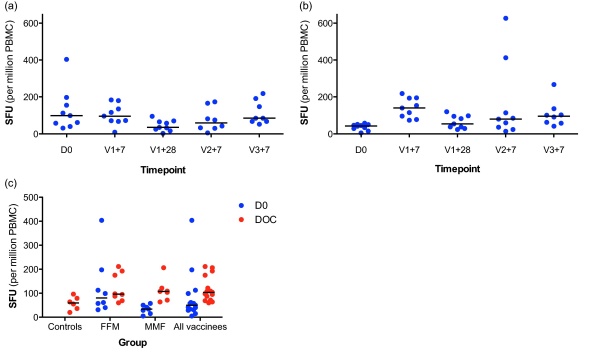
Prime-boost groups immunogenicity to L3SEPTL by timepoint. (a) FFM (group 6); (b) MMF (group 7); (c) vaccinees and controls pre-challenge. Summed *ex vivo* IFNγ-ELISPOT responses to whole L3SEPTL insert at various timepoints. ‘DOC’ = day of challenge (V3+14 days). Sample numbers pre-challenge are as stated in Fig. 6 and at DOC *n* = 8, 7 and 6 for FFM, MMF and control groups respectively. Horizontal lines represent the median.

**Fig. 6:**
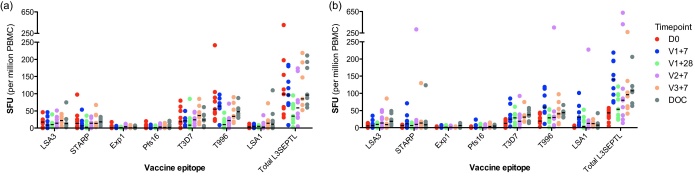
Prime-boost groups immunogenicity to vaccine antigens. (a) FFM (group 6) and (b) MMF (group 7). *Ex vivo* IFNγ-ELISPOT responses to individual vaccine antigens or the whole vaccine insert (‘Total L3SEPTL’) at baseline (‘D0’), 7 days following each vaccine (‘V1+7’, ‘V2+7’ and ‘V3+7’) and at 28 days following vaccine 1 (‘V1+28’). Results are summed from responses against multiple pools of peptides where applicable. For FFM, *n* = 9 (D0, V1+7 and V1+28) or 8 (V2+7 and V3+7). For MMF, *n* = 10 (D0), 9 (V1+7, V1+28 and V2+7) or 8 (V3+7). Horizontal lines represent the median. T996 and T3D7 refer to TRAP sequences from the vaccine (T9/96) and challenge (3D7) strains of *Plasmodium falciparum*.

IFNγ ELISPOT responses against vaccine antigens for the prime-boost groups are shown in Figs. 5 and 6. Overall, vaccine immunogenicity was lower than expected based on studies of other malaria antigens in the same poxvirus vectors [7,21,22]. Median responses to the whole vaccine insert (L3SEPTL) at seven days after the last vaccine (V3+7) were 85 (IQR 68–180) and 96 (59–128) sfu/10^6^ PBMC for the FFM and MMF groups respectively compared to a pre-vaccination response of 80 (44–176) and 37.5 (18–49) respectively (Fig. 5). This was a statistically significant increase for the MMF group (Wilcoxon's matched pairs test, *p* = 0.008). Pre-vaccination responses to the vaccine insert for the FFM group were unexpectedly high in comparison to the MMF group. These responses were mainly directed against TRAP from the parasite strain used in the vaccine insert (T9/96) and were significantly higher than those in the MMF group (Mann–Whitney test, *p* = 0.003). This is unlikely to be a laboratory error as clinical procedures and laboratory assays for both groups occurred concurrently and laboratory staff were blinded to volunteer group assignment.

MVA-PP induced a statistically significant priming response (of 140 sfu/million PBMC) to the whole L3SEPTL insert in the MMF group (Wilcoxon's matched pairs test, *p* = 0.008) where FP9-PP failed to do so in the FFM group (*p* = 0.68) when comparing pre-vaccination responses with those at V1+7. There was no significant rise in responses after the second vaccination (Wilcoxon's matched pairs test, *p* = 0.67 for FP9-PP and *p* = 0.31 for MVA-PP at V2+7 compared to V1+28 for the FFM and MMF groups respectively). However, MVA-PP again induced a significant rise in responses to L3SEPTL at the final (boosting) dose (Wilcoxon's matched pairs test, *p* = 0.04 for MVA-PP, *p* = 0.67 for FP9-PP for the FFM and MMF groups respectively, comparing V3+7 with V2+7 in each case).

Responses were more frequently identified and stronger to the four larger antigens, LSA3, LSA1, TRAP and STARP than to the smaller Exp1 and Pfs16 (Fig. 6) but peptide pools from all antigens were recognised by at least one vaccine.

There was a small rise in non-malaria-specific background IFNγ responses (to culture medium alone) after the first vaccination with MVA-PP at low dose (1 × 10^8^ pfu). Median responses were 3.75 and 11.25 sfu/10^6^ PBMC at baseline (D0) and 7 days after vaccine 1 (V1+7) respectively (Wilcoxon's matched pairs test, *p* = 0.003, *n* = 12) (see Online Fig. A).

### 3.5 No clinical protection against *P. falciparum* sporozoite challenge

Fifteen vaccinees underwent *P. falciparum* sporozoite challenge two weeks after receiving their final immunisation. Six unvaccinated, malaria-naïve volunteers also took part to confirm the effectiveness of the challenge model. The procedure was well-tolerated and there were no SAEs recorded. A total of 19 AEs were recorded in 13 (61.9%) challenges over four weeks following the challenge. One was judged of moderate severity (fatigue) but the rest were judged mild. One volunteer developed a transient localised petechial rash on one forearm after the application of a tourniquet. Mild thrombocytopenia was noted (platelet count 114 × 10^9^/L) which resolved without intervention.

Expected symptoms of malaria were not recorded as AEs and included anorexia, chills, diarrhoea, fever, headache, low back pain, myalgia or arthralgia, nausea or vomiting, rigors and sweats. One or more of these symptoms was recorded in 80% of vaccinees and in 100% of unvaccinated controls. Although all symptoms were more frequent in the control group than vaccinees this is of unknown significance in this unblinded study.

All 21 volunteers developed detectable parasitaemia by thick film microscopy during the 21-day surveillance period and were treated with anti-malarial medication without any significant complication. All volunteers also developed positive PCR tests for malaria parasites during the follow up period.

### 3.6 No delay in time to parasitaemia

**Fig. 7:**
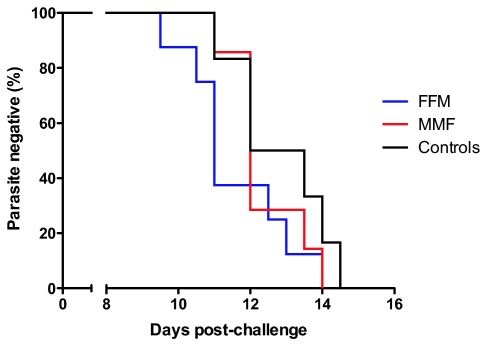
Kaplan–Meier survival curve. Time to parasitaemia is plotted for vaccinees in FFM (*n* = 8) or MMF (*n* = 7) groups and unvaccinated control volunteers (*n* = 6). Log-rank (Mantel–Cox) test for difference in curves: *p* = 0.35.

All vaccinees were diagnosed with blood-film positive malaria by the morning of day 14 and all control volunteers by the evening of day 14 following challenge. The mean day of diagnosis for all vaccinees was 11.9 compared to 12.8 for control volunteers. There was no significant difference between the curves representing time to slide positivity (Fig. 7, Log-rank Mantel–Cox test, *p* = 0.35) or mean time to diagnosis between the FFM group, MMF group or all vaccinees compared to controls (Table 2, Mann–Whitney test, *p* = 0.13, 0.55 and 0.20 respectively). There was also no significant correlation between the magnitude of the ELISPOT response on the day of challenge (DOC) and the time to blood-film positive malaria in either vaccine regime or all vaccinees together (Spearman's correlation, data not shown).

### 3.7 No reduction in blood stage parasite growth rate by PCR

Serial quantitative PCR measurements to detect malaria parasite DNA were carried out up to twice daily during the trial to estimate blood stage parasite growth rates over the challenge period for each volunteer. Clinic staff and laboratory staff responsible for blood film assessment were blinded to these results until after anti-malarial treatment.

The vaccines used in this study were designed to elicit pre-erythrocytic cellular responses primarily. However, differences in the growth rate of the parasite asexual blood stages between vaccinees and controls would suggest a specific blood stage effect of vaccination. The same growth rate data can also be used to derive information on pre-erythrocytic efficacy by back-calculating parasite numbers to the day of emergence into the blood. Thus an estimate of the number of infected hepatocytes responsible for the emerging merozoite load can be calculated for each volunteer. A reduction in this number would suggest a pre-erythrocytic effect of vaccination, even if insufficient to prevent eventual parasitaemia.

**Fig. 8:**
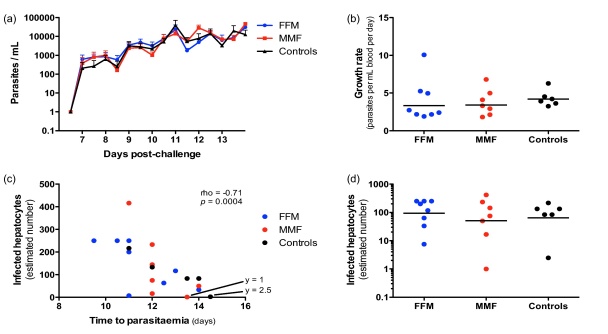
PCR results and estimations. (a) Parasite density during challenge follow up. (b) Mean parasite growth rate estimated from PCR data. (c) Infected hepatocyte numbers estimated from PCR data correlate with time to parasitaemia. The annotations (‘*y* = ’) give the number of infected hepatocytes for points close (but not equal to) zero. (d) Data shown in (c) but separated by study group. Error bars in (a) are the standard error of the mean and horizontal lines in (b) and (d) represent the geometric mean.

Various methods for estimating growth rates exist, including simple linear estimation, a sine wave approximation [23] and a statistical model method [20]. We employed the statistical method in this study. Although mean blood stage parasite growth rates were lower in the vaccinated groups (Fig. 8), this was not statistically significant, even when vaccine groups were analysed together (*p* = 0.29), suggesting that any blood stage effect of vaccination was minimal. Asexual blood stage growth rates did not correlate significantly with time to parasitaemia (data not shown). However, the estimated number of infected hepatocytes generated during the liver stage of infection (derived from the PCR rate data) does correlate with the time to blood-film positive parasitaemia (Spearman's *p* = 0.0004, rho = −0.71, Fig. 8c).

## 4 Discussion

### 4.1 Rationale

We conducted a prospective phase I/IIa dose-escalation and sporozoite challenge trial in healthy malaria-naïve human volunteers administered the novel malaria vaccines FP9-PP and MVA-PP. Vaccinations in the prime-boost groups were given one month apart and volunteers underwent challenge three weeks after the last vaccination.

The vaccines encode a ‘polyprotein’ construct (‘L3SEPTL’) consisting of six pre-erythrocytic malaria antigens (from N to C terminus): LSA3, STARP, Exp1, Pfs16, TRAP and LSA1. Although the aim of immunisation was to stimulate a pre-erythrocytic cellular response, expression during the blood stage of the malaria parasite lifecycle has also been reported for STARP [13], Exp1 [14] and for a LSA3 homologue [12,24]. Pfs16 is also expressed at sexual stages [25]. The expressed protein is 3240 amino acids long and has been shown to induce T cell responses to peptide pools from each of the six antigens in mice [4]. To our knowledge this is the largest foreign insert in a viral vectored vaccine tested in a clinical trial.

The viral vectors employed here have been used extensively in human vaccination [7,26,27]. Previous vaccine studies using these vectors in human prime-boost regimes with much smaller inserts have demonstrated the ability to induce strong T-cell responses measured by the *ex vivo* IFNγ-ELISPOT and induce sterile protection on malaria challenge in some volunteers [7]. The approach explored in this study was to attempt to broaden the vaccine-induced immune response to cover multiple malarial antigens and provide strong pre-erythrocytic and perhaps some blood-stage immunity. The potential advantages of a broader immune response should be to: (1) reduce the risk of immune escape; (2) improve potential protective efficacy by increasing the number of antigens and epitopes targeted by protective T cells; (3) limit inter-individual variation in vaccine immunogenicity related to HLA-restriction and lack of T cell epitopes in a single antigen insert; and (4) provide a more cost-effective solution than vaccinating with mixtures of multiple single-antigen vaccines.

### 4.2 Safety

Both vaccines were found to be safe and well tolerated. Higher doses of the vaccines did not appear to increase the frequency or severity of local AEs. Increasing doses of MVA-PP were associated with a greater frequency of systemic AEs, though generally of mild severity. Successive doses of vaccine in the MMF regime led to a detectable increase in the severity of local AEs but no clear effect on AE duration.

### 4.3 Cellular immunity

IFNγ ELISPOT responses to single vaccine doses were low. There was no clear effect of dose on immune response in the dose-escalation groups, but these group sizes were not powered to allow immunogenicity comparisons, and responses were expected to be low following a single priming dose.

However, immunogenicity was also disappointingly low in the two heterologous prime-boost groups. FP9-PP failed to induce a significant priming response in the FFM group (albeit from a relatively high baseline) but also failed to boost responses in the MMF group. Median responses in the MMF group reached only 140 sfu/million PBMC following priming compared to 43 sfu/million PBMC at baseline. In comparison, previous prime-boost vaccine studies using these vectors expressing the TRAP antigen have yielded up to 400–500 sfu/10^6^ PBMC [7,21]. Where partial protection was achieved, with an ME-TRAP insert, the magnitude of peak immunogenicity correlated with delay to parasitaemia [7], indicating that responses in the present study were very unlikely to have reached protective levels. Previous work with FP9-PP and MVA-PP in mice [4] examined the CD8 response primarily after intravenous administration of vaccine and is not easily comparable, particularly as human immunogenicity with many vaccines is often lower than that observed in murine studies.

The reasons for this failure of immunogenicity are uncertain. Possible explanations include: (1) the size of the L3SEPTL protein produced may have limited expression of the transgene so that insufficient protein was produced to induce a strong immune response. The polyprotein used here is substantially larger than others reported to date and was under the control of a standard poxvirus p7.5 promoter. (2) The large number of potential epitopes present in the polyprotein construct may have resulted in significant competition between antigens all of which are expressed in the same cell. (3) Increasing evidence supports cross-priming as the principal method of presentation of antigens expressed by poxviruses [28], although the extent to which this mechanism can allow immunogenicity of large complex inserts is unclear.

Importantly, none of these suggested mechanisms prevented immunogenicity of the same vaccine vectors in murine studies [4]. While this may represent a dose effect related to the relatively greater dose per weight administered in mice, it could also suggest that any effect of insert size may be greater in humans than in mice. Further studies will be required to assess the effects of dose and limits of transgene size that can be effectively expressed in poxvirus vaccines in humans and to assess relevant mechanisms.

### 4.4 Efficacy

The vaccine regimes studied here were unable to induce sterile protection in a sporozoite challenge or delay the onset of patent parasitaemia in vaccinees. IFNγ-ELISPOT responses at challenge did not predict how quickly parasitaemia developed. Estimates of infected hepatocyte numbers responsible for subsequent blood-stage parasite load and growth in vaccinees proved to be a good predictor of time to slide positive parasitaemia across all challenged subjects. This study was designed to assess a possible liver stage effect of vaccination, but if a significant blood stage effect had been anticipated then a blood stage challenge protocol [29] may have been preferable.

## 5 Conclusions

There is an increasing consensus in the malaria vaccine development field that multiple antigens will be required in a vaccine to achieve high levels of efficacy in field trials. Heterologous prime-boost immunisation has been one of the very few approaches to successfully induce sterile efficacy in any human vaccinees and this study has assessed a polyprotein approach to broadening the immunogenicity of the induced T cell responses. Our results suggest that there may be limits to the insert size that will be readily immunogenic in humans, at least using standard vaccinia promoters. Hence other vector design strategies, such as the use of multiple promoters and insertion sites [30], or mixtures of single vaccines may be more suitable for exploiting the great capacity of poxviruses to express foreign antigens.

## Figures and Tables

**Table 1:** Volunteer demographics.

Group	*N*	Mean age (SD)	Min age	Max age	No. female (%)	No. male (%)
G1: low dose F	3	35.4 (8.7)	27.9	45.0	2 (66.7%)	1 (33.3%)
G2: low dose M	3	22.4 (2.8)	19.3	24.6	3 (100%)	0 (0%)
G3: mid dose F	3	34 (6.4)	27.3	40.0	2 (66.7%)	1 (33.3%)
G4: mid dose M	3	28.8 (11.5)	21.5	42.1	0 (0%)	3 (100%)
G5: high dose M	3	29.5 (13.8)	18.9	45.1	1 (33.3%)	2 (66.7%)
G6: FFM	9	32.2 (10.5)	20.6	47.3	6 (66.7%)	3 (33.3%)
G7: MMF	10	29.8 (7.5)	19.5	44.6	3 (30%)	7 (70%)
Total cohort	34	30.5 (8.9)	18.9	47.3	17 (50%)	17 (50%)

Data is for all volunteers enrolled (vaccinated) in the study. Age is given in decimal format. Data on subjects’ ethnic origin was not collected.

**Table 2:** Time to diagnosis for vaccinees in FFM and MMF groups and unvaccinated control volunteers.

Group	Number in group	No. positive for parasites by day 21	Mean time to diagnosis (days)	SD	Median day of diagnosis	Interquartile range
Group 6	8	8	11.6	1.5	11	10.9–12.6
Group 7	7	7	12.4	1.0	12	12–12.8
All vaccinees	15	15	11.9	1.3	12	11–12.8
Controls	6	6	12.8	1.4	12.8	12–13.9
