# Supplementary material for: A human Phase I/IIa malaria challenge trial of a polyprotein malaria vaccine
Source: Vaccine. 2011 Oct 6;29(43):7514–22. doi: 10.1016/j.vaccine.2011.03.083 (PMC3195259; doi:10.1016/j.vaccine.2011.03.083)
Supplement: Supplementary file 1 [file mmc1.pdf]

Supplemental data

Grade	Redness (mm)	Swelling (mm)	Pain	Other
0	0	0	No pain at all	None
1	< 50	< 20	Painful to touch. No restriction in movement of arms, able to work, drive, carry heavy objects as normal	Mild: Transient or mild discomfort (< 48 hours); no medical intervention/therapy required
2	50 – 100	20 – 50	Painful when limb is moved (*i.e.* restriction in range of movement in arm, difficulty in carrying objects)	Moderate: Mild to moderate limitation in activity - some assistance may be needed; no or minimal medical intervention/therapy required
3	> 100	> 50	Severe pain at rest (*i.e.* unable to use arm due to pain.)	Severe: Marked limitation in activity, some assistance usually required; medical intervention/therapy required, hospitalisation possible
4	-	-	-	Serious, life-threatening: Extreme limitation in activity, significant assistance required; significant medical intervention/therapy required, hospitalisation or hospice care probable

Online Table A: **Severity grading scales** used to assess adverse events.

(a)

Description	**Group 1:** FP9-PP low dose No. volunteers (%) (n = 3)			**Group 2:** MVA-PP low dose No. volunteers (%) (n = 3)			**Group 3:** FP9-PP mid dose No. volunteers (%) (n = 3)			**Group 4:** MVA-PP mid dose No. volunteers (%) (n = 3)			**Group 5:** MVA-PP high dose No. volunteers (%) (n = 3)		
	**Mild**	**Mod**	**Severe**	**Mild**	**Mod**	**Severe**	**Mild**	**Mod**	**Severe**	**Mild**	**Mod**	**Severe**	**Mild**	**Mod**	**Severe**
Itch	3 (100%)			2 (67%)			3 (100%)			2 (67%)			3 (100%)		
Pain	3 (100%)			3 (100%)			2 (67%)	1 (33%)		2 (67%)	1 (33%)		2 (67%)		
Redness	2 (67%)	1 (33%)		3 (100%)			3 (100%)			3 (100%)			2 (67%)	1 (33%)	
Scaling	3 (100%)			3 (100%)			3 (100%)			3 (100%)			3 (100%)		
Swelling	2 (67%)	1 (33%)		2 (67%)	1 (33%)		1 (33%)	2 (67%)		2 (67%)		1 (33%)	1 (33%)	2 (67%)	
Warmth	3 (100%)			2 (67%)			3 (100%)			2 (67%)			3 (100%)		
**Total**	**16**	**2**	**0**	**15**	**1**	**0**	**15**	**3**	**0**	**14**	**1**	**1**	**14**	**3**	**0**
Arthralgia	1 (33%)						1 (33%)						1 (33%)		
Documented fever	1 (33%)						1 (33%)						1 (33%)		
Fatigue	3 (100%)									2 (67%)			3 (100%)		
Feverish	2 (67%)						1 (33%)			2 (67%)			3 (100%)		
Headache	2 (67%)			1 (33%)			1 (33%)			3 (100%)					
Malaise	2 (67%)									2 (67%)			1 (33%)		
Myalgia	3 (100%)			1 (33%)			1 (33%)			1 (33%)			2 (67%)		
Nausea										1 (33%)			1 (33%)		
**Total**	**14**	**0**	**0**	**2**	**0**	**0**	**5**	**0**	**0**	**11**	**0**	**0**	**12**	**0**	**0**

(b)

**Description**	**Group 6 (FFM)**									**Group 7 (MMF)**								
	**Vaccine 1** No. volunteers (%) (n = 9)			**Vaccine 2** No. volunteers (%) (n = 9)			**Vaccine 3** No. volunteers (%) (n = 8)			**Vaccine 1** No. volunteers (%) (n = 10)			**Vaccine 2** No. volunteers (%) (n = 9)			**Vaccine 3** No. volunteers (%) (n = 8)		
	**Mild**	**Mod**	**Severe**	**Mild**	**Mod**	**Severe**	**Mild**	**Mod**	**Severe**	**Mild**	**Mod**	**Severe**	**Mild**	**Mod**	**Severe**	**Mild**	**Mod**	**Severe**
Itch	7 (78%)			5 (56%)			6 (75%)			9 (90%)			9 (100%)			7 (88%)		
Pain	7 (78%)	1 (11%)		6 (67%)	1 (11%)		7 (88%)	1 (13%)		8 (80%)			7 (78%)			5 (63%)	3 (38%)	
Redness	7 (78%)	2 (22%)		4 (44%)	5 (56%)		4 (50%)	4 (50%)		9 (90%)	1 (10%)		6 (67%)	3 (33%)		4 (50%)	4 (50%)	
Scaling	7 (78%)			9 (100%)			6 (75%)			10 (100%)			8 (89%)			6 (75%)		
Swelling	6 (67%)	3 (33%)		6 (67%)	3 (33%)		4 (50%)	4 (50%)		8 (80%)	2 (20%)		5 (56%)	3 (33%)		3 (38%)	5 (63%)	
Warmth	7 (78%)			7 (78%)			8 (100%)			7 (70%)			7 (78%)			7 (88%)		
**Total**	**41**	**6**	**0**	**38**	**9**	**0**	**35**	**9**	**0**	**51**	**3**	**0**	**42**	**7**	**0**	**32**	**12**	**0**
Arthralgia	3 (33%)			4 (44%)			2 (25%)			2 (20%)			1 (11%)			4 (50%)		
Documented fever	2 (22%)			2 (22%)						2 (20%)						1 (13%)	2 (25%)	
Fatigue	3 (33%)			4 (44%)			2 (25%)			1 (10%)			2 (22%)			2 (25%)		
Feverish	2 (22%)			4 (44%)			2 (25%)			5 (50%)			1 (11%)			6 (75%)		
Headache	2 (22%)	1 (11%)		3 (33%)			2 (25%)			3 (30%)			3 (33%)			5 (63%)		
Malaise	2 (22%)			3 (33%)			3 (38%)			3 (30%)			1 (11%)			5 (63%)		
Myalgia	3 (33%)			6 (67%)			4 (50%)			5 (50%)			2 (22%)			5 (63%)		
Nausea							1 (13%)			1 (10%)			1 (11%)			2 (25%)		
Other													1 (11%)					
**Total**	**17**	**1**	**0**	**26**	**0**	**0**	**16**	**0**	**0**	**22**	**0**	**0**	**11**	**0**	**0**	**30**	**2**	**0**

Online Table B: **Frequency of vaccine-related AEs.** The number and proportion of volunteers reporting an AE at least once within the first month following vaccination is shown for **(a) single dose groups** and **(b) prime-boost groups**

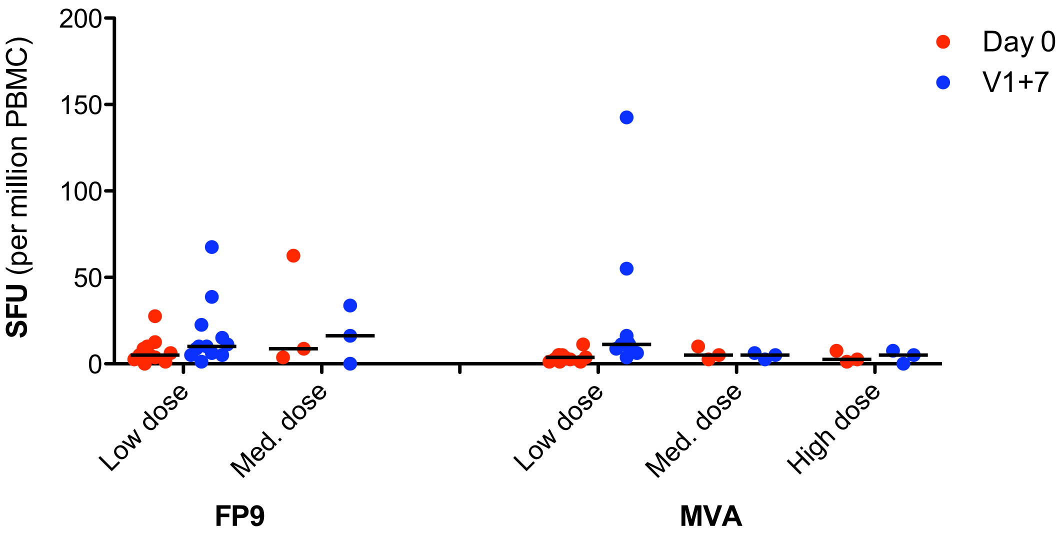

Online Figure A: **Non-specific responses.** *Ex-vivo* IFNγ-ELISPOT responses to culture medium alone at baseline and 7 days following the first vaccination ('D0' and 'V1+7' respectively). Responses are displayed as spot forming units (sfu) per million PBMC. Horizontal lines represent the median.
